# Expanding the roles of malaria post workers in Thailand: A qualitative study of stakeholder perspectives

**DOI:** 10.1371/journal.pgph.0003670

**Published:** 2024-09-17

**Authors:** Monnaphat Jongdeepaisal, Panarasri Khonputsa, Orathai Prasert, Supitsara Maneenate, Massaya Sirimatayanant, Paradorn Sopa, Arisa Saisong, Ittisak Charoensup, Tanong Kamsri, Rungrawee Tipmontree, Prayuth Sudathip, Marco Liverani, Richard J. Maude, Christopher Pell

**Affiliations:** 1 Mahidol Oxford Tropical Medicine Research Unit, Faculty of Tropical Medicine, Mahidol University, Bangkok, Thailand; 2 Centre for Tropical Medicine and Global Health, Nuffield Department of Medicine, University of Oxford, Oxford, United Kingdom; 3 Ubon Ratchathani Provincial Health Office, Ubon Ratchathani, Thailand; 4 Sisaket Provincial Health Office, Sisaket, Thailand; 5 Buntharik Hospital, Buntharik, Ubon Ratchathani, Thailand; 6 Phibun Mangsahan Hospital, Phibun, Ubon Ratchathani, Thailand; 7 Division of Vector Borne Diseases, Department of Disease Control, Ministry of Public Health, Nonthaburi, Thailand; 8 Department of Global Health and Development, London School of Hygiene and Tropical Medicine, London, United Kingdom; 9 School of Tropical Medicine and Global Health, Nagasaki University, Nagasaki, Japan; 10 Faculty of Public Health, Mahidol University, Bangkok, Thailand; 11 The Open University, Milton Keynes, United Kingdom; 12 Amsterdam Institute for Global Health and Development (AIGHD), Amsterdam, The Netherlands; 13 Department of Global Health, Amsterdam University Medical Centers, Academic Medical Center, Amsterdam, The Netherlands; 14 Centre for Social Science and Global Health, University of Amsterdam, Amsterdam, The Netherlands; ICMR-National Institute of Malaria Research: National Institute of Malaria Research, INDIA

## Abstract

In Thailand, since the 2000s, malaria post (MP) workers have been tasked with promptly detecting and treating all malaria cases to prevent onward transmission in the communities. Expanding their roles to provide health services beyond malaria has been proposed as a strategy to sustain their activities until elimination is reached. This article examines the perspectives of stakeholders on community-based malaria care to assess prospects for expanding the role of MPs. The study incorporated in-depth interviews (IDIs) and focus group discussions (FGDs). In forested communities and local health facilities in northeast Thailand bordering Lao PDR and Cambodia, where malaria transmission is low, IDIs were conducted with 13 MPs and 23 community members. An additional 14 policymakers and implementers across the health sector in Thailand were interviewed. The respondents highlighted how in these border areas population groups most at risk of malaria, namely forest goers and migrants, are reluctant to visit public health facilities. In these areas, MP workers are well integrated in their communities and remain relevant although the communities no longer see malaria as spriority. Common conditions such as dengue, diabetes, insect bites, diarrhea, mental illness and substance abuse, were identified as local health concerns needing potential add-on services from MP workers. Although challenges in terms of training, supervision, and financing were raised, opportunities included additional funds from local administrative offices to maintain and integrate malaria activities with other health programmes. Changes to the roles of MPs should be adapted to changing local needs, some of which were identified in this study, should avoid duplication and potential tensions with other local health programmes, and need to build on the capacity of the community and primary care system. These enabling factors are worthy of consideration by any malaria programmes looking into maintaining their village malaria workers in the Greater Mekong Subregion.

## Introduction

Despite progress towards malaria elimination in the Greater Mekong Subregion (GMS), the disease remains a public health threat [1]. It is particularly acute in remote rural areas, where access to health care is limited and where communities bear the greatest health and economic burden [2]. Many of these communities suffer from shortages of health professionals and lack of infrastructure [3].

Accessible community-based malaria services are essential to reach at-risk populations, particularly forest goers and mobile and migrant groups. The introduction of rapid diagnostic tests (RDTs) for malaria in the early 2000s enabled an expansion of the role of village malaria workers (VMWs). In many malaria endemic areas, VMWs play a pivotal role in ensuring early diagnosis and treatment in the community [4, 5]. In Thailand, various programmes involving community-based malaria control activities have been established since the 1960s. These include malaria volunteers (1965) and village health volunteers (1977) under the supervision of health promoting hospitals (HPH) within the general public health system. Malaria clinics (MCs) (1979) have also been established under a vertical programme run by the Division of Vector-Borne Diseases (DVBD) to provide early diagnosis and treatment [6]. Since 2004, malaria post (MP) workers have been recruited and managed by the Ministry of Public Health, receiving support from international organisations, primarily The Global Fund to Fight AIDS, Tuberculosis and Malaria [7].

As malaria cases decline to almost zero, there is a risk that these services are scaled back, which could hinder elimination. One approach to increase the sustainability of community-based malaria services is to expand the roles of VMWs. In Thailand (and elsewhere), the success of this approach requires an understanding of policy challenges and opportunities at different levels of the health system. However, there is limited understanding of how expanding the roles of MPs (and VMWs more broadly) could align with malaria elimination priorities and the current public health needs in endemic communities, as well as how MPs could be leveraged to achieve these goals. Drawing on in-depth interviews (IDIs) and focus group discussions (FGDs) with stakeholders (including policy makers/implementers, healthcare staff and members of malaria endemic communities) in Thailand, this article examines prospects for, and challenges to, expanding the roles of MPs.

## Methods

### Ethics statement

Ethical approval was obtained from the Oxford Tropical Research Ethics Committee (OxTREC Reference: 535–21, 24 August 2021; 552–21, 22 September 2021) and the Institute for the Development of Human Research Protections (IHRP) at the Health Systems Research Institute (HSRI) (IHRP reference: 146–2564, 30 September 2021; 169–2564, 18 November 2021). All respondents provided written informed consent and were asked for consent to be audio-recorded. Respondents were informed that they could withdraw from the study at any time, with no consequences for them.

The study team was granted permission from DVBD and PHOs to conduct research activities at the study sites. DVBD, PHOs, and local health facilities were consulted throughout the data collection period.

### Study design

This study was part of a multidisciplinary, multi-country project (2021–2023), comprising a systematic review of recent malaria community health worker programmes in the Asia-Pacific, a scoping survey of national malaria control programmes (NMCPs) and malaria programme managers, implementer interviews and a survey in malaria endemic communities [8]. This project was supported by the Regional Artemisinin Initiative 3 Elimination (RAI3E), a large programme funded by The Global Fund to Fight AIDS, Tuberculosis and Malaria to accelerate elimination of *Plasmodium falciparum* malaria in the GMS [9]. Here the results of the rapid policy document review and stakeholder qualitative interviews are presented.

### Settings

**Fig 1:**
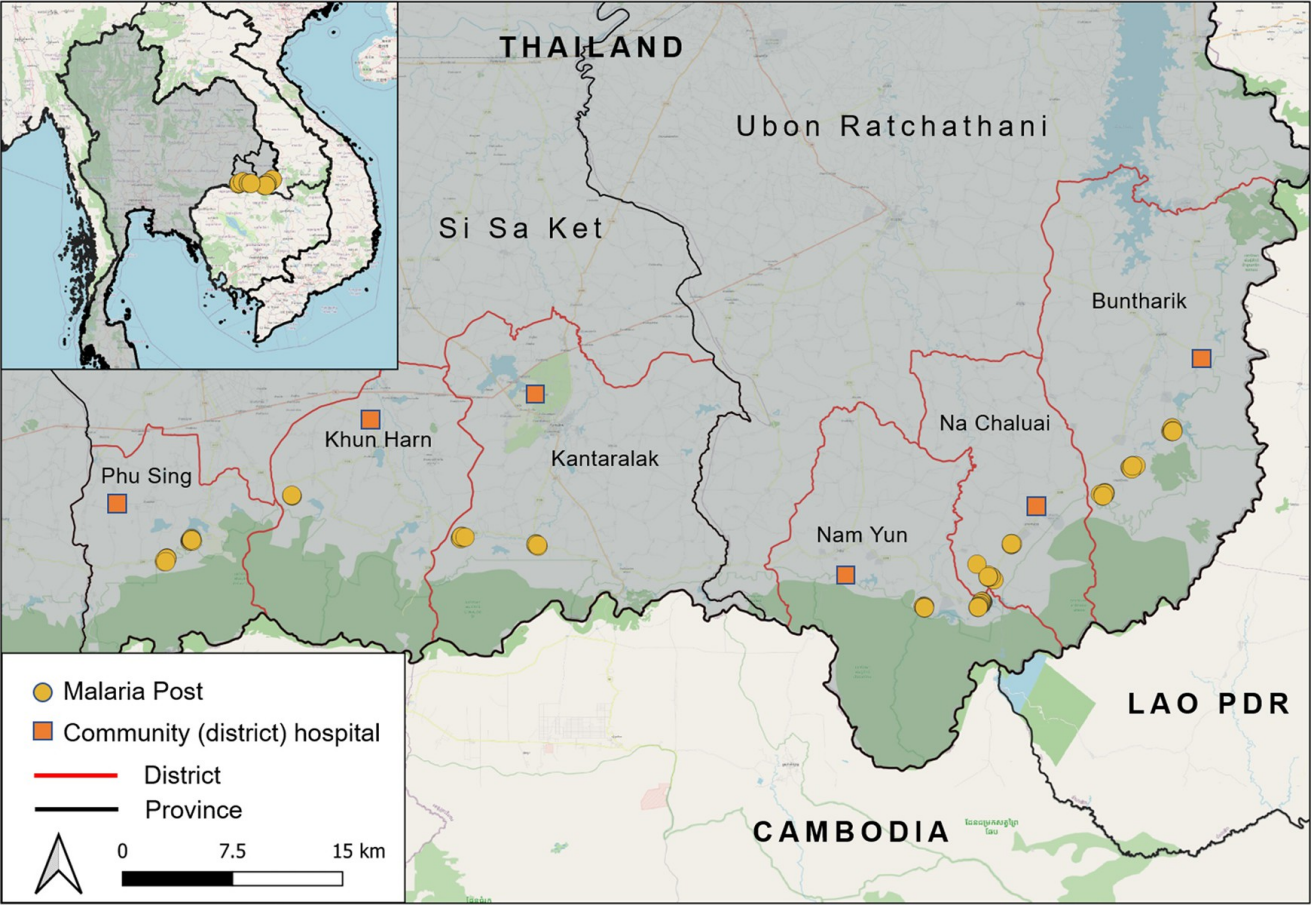
Study provinces and locations of study villages along the Thailand-Lao PDR-Cambodia border. The map was created using QGIS software version 3.26 (Buenos Aires) and contains information from OpenStreetMap and OpenStreetMap Foundation, which is made available under the Open Database License. National and provincial administrative boundaries were from Global Administrative Areas version 3.6 (https://gadm.org/download_country_v3.html). GPS locations of the study sites were collected by the authors using KoboToolbox.

**Fig 2:**
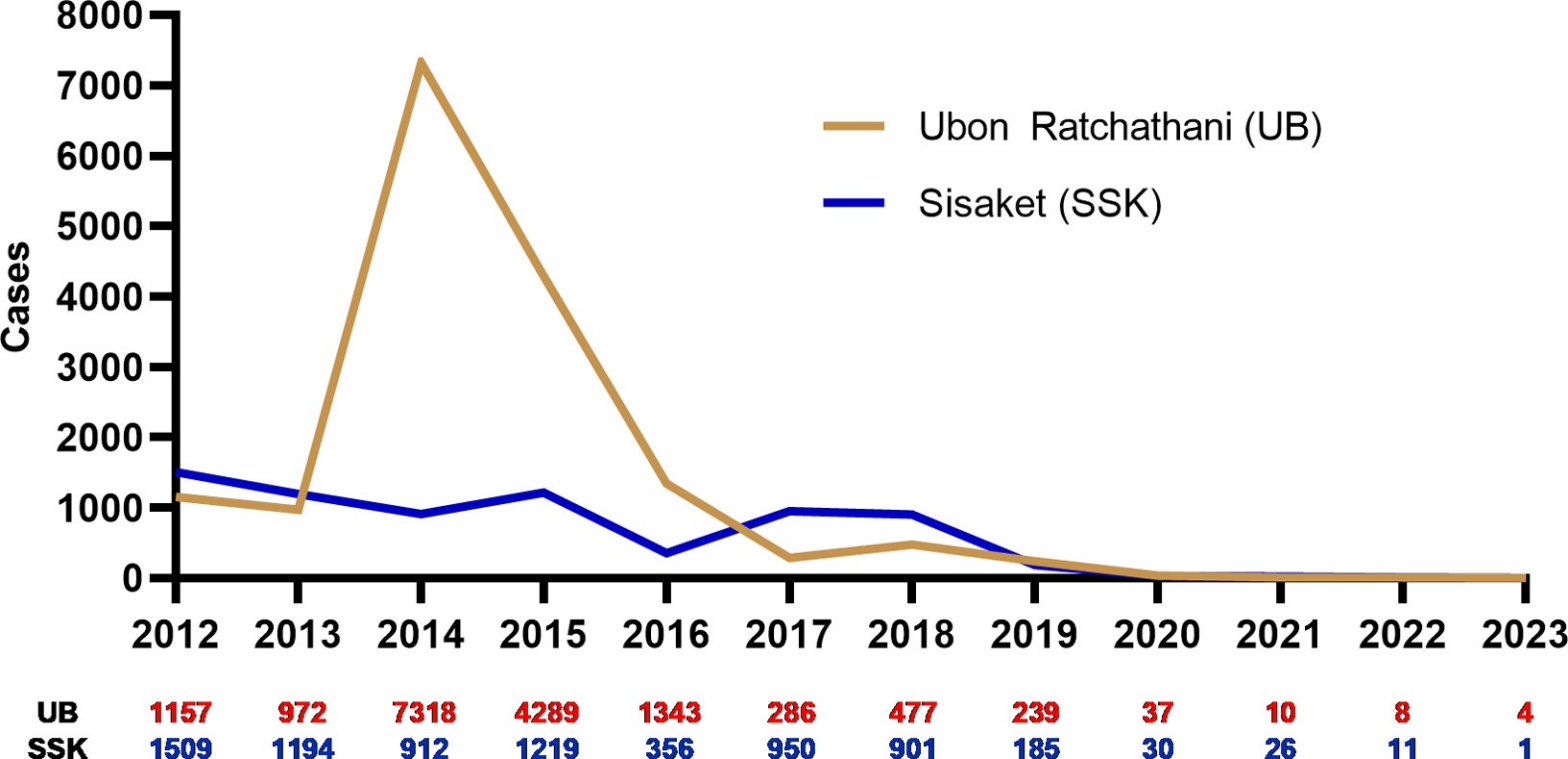
Number of malaria cases in Ubon Ratchathani and Sisaket provinces, 2012–2023. The figure was created using reported numbers of malaria cases during 2012–2023 from the Thai Ministry of Public Health: https://malaria.ddc.moph.go.th/malariar10/index_newversion.php.

Sites for the interviews with community stakeholders and MPs were identified in consultation with the Thailand NMCP within the Division of Vector-Borne Diseases (DVBD). Ubon Ratchathani and Sisaket provinces in northeast Thailand were selected because they were endemic communities with low malaria transmission and near to elimination. The study team also had an ongoing collaboration with the Provincial Health Offices (PHO) and had conducted previous studies in the area [10]. In these provinces, we purposively selected malaria endemic communities with active MPs, located along the Thai-Lao-Cambodian border ‐ the regional and global epicenter of artemisinin resistance [11] (see **Fig 1**). This area recorded Thailand’s highest malaria incidence rate during an outbreak in 2014 (see **Fig 2**), but this has greatly reduced since [12].

According to DVBD’s RAI3E strategy plan for 2021–2023 [7], in 2021 there were 8 MCs, 13 MPs, and 46 HPHs (also known as health centers or primary care units) in these two provinces that provided malaria services. These providers are primarily located in zones A1 (defined as active foci areas with indigenous cases in the current year) and A2 (defined as residual non-active foci areas with no indigenous cases in the previous 1–3 years). Nationally, there were 180 MCs, 400 MPs and 444 HPHs located in 40 provinces with endemic malaria in 2021. The malaria zones and implementation areas under the RAI3E initiative are reviewed and updated every year.

### Respondents and recruitment

Respondents included MPs, adult members of the malaria endemic communities, policy makers and implementers. In the selected study sites, MPs were purposively identified based on a contact list generated during past projects and by the PHO staff who supervised them. At the time of data collection, there were 13 MPs, each living in the 13 endemic communities in the two provinces, and all were invited to an interview. Adult community members (≥18 years old) were identified through consultations with MPs and selected if they were regularly exposed to malaria risk due to their work in the forest or if they had visited MPs for their services. Snowball sampling was utilized to recruit respondents from particular groups, such as pregnant women, elderly, families with children, and local community leaders. Community members were approached at their homes. Similar criteria were applied for the selection of focus group participants, who were invited to join the group discussion at a participant’s house or other village house in the communities.

Policymakers and implementers were identified through consultation with DVBD and a rapid document review. Published policy documents and articles in Thai and English languages were selected for review to extract preliminary information regarding the set up and key implementers of Thailand’s malaria programme and health volunteer programmes, including MPs and VHVs. Eligible respondents included governmental health-sector managers at the central and provincial levels, researchers with expertise in community-based care, representatives of international organizations, and implementers in non-governmental organizations. Healthcare workers and public health staff in HPHs and public hospitals, including medical doctors and nurses, were also invited to join the interviews. They were approached by email, phone call, or in-person at their place of work.

### Data collection tools

Given the exploratory nature of this study, interview schedules were lightly structured, tailored to the role and expertise of each informant, and refined in light of emerging findings. The interviews aimed to elicit perspectives and expectations about present and future MP services, and the resources needed to implement the expanded services effectively. The FGDs explored these topics in a group setting; specifically what the local health concerns were and how the expanded MP roles could be implemented and developed to better reach specific population groups in the communities such as the elderly and forest goers, and work arrangements for MPs. The topic guides were developed based on the results from the rapid review and from our previous study of forest malaria and at-risk groups in Thailand [10] (see **S1 Appendix** for IDI and FGD guides). The topic guides were initially drafted in English, translated into Thai, and reviewed for clarity and correct translation. The guides were flexibly used to direct conversations, and were updated using insights from earlier interview responses. For example, questions about MPs performing roles beyond malaria were updated during data collection to further explore emerging findings and capture the extensive nature of volunteer work in community settings. Emerging themes around key topics, such as the horizontal integration strategy and border malaria, were triangulated with subsequent interviews with policymakers and implementers.

### Data collection

Data collection activities were conducted from 19 November 2021 to 16 June 2022 by four female researchers and research assistants (MJ PK OP and SM) involved in this study. Interviews with participants were conducted in a quiet location at their residence, communal place, or place of work. Interviews with community members and healthcare workers were conducted in Thai and Thai-Isan (northeastern) languages at the study sites. Interviews with policymakers and implementers were conducted in either Thai or English, in-person and/or via an online platform. In-depth interviews lasted from 30 minutes to 2 hours. With the consent of respondents, interviews and FGDs were digitally recorded.

### Data analysis

Audio recordings were transcribed verbatim by two research assistants (OP and SM), and, if necessary, translated into English by one social scientist (MJ). The translated transcripts and interview notes were imported into NVivo version 12 (QSR International) for qualitative thematic analysis [13]. All transcripts were coded line-by-line. The codebook was initially created based on the interviews or discussion topics and, subsequently, during the process of coding, sub-themes that emerged from the data were incorporated into the codebook. Interim outputs such as stakeholder matrix tables [14, 15] and local community network maps were constructed to better understand the roles of different stakeholders, their resources, and their views on the expansion of roles.

## Results

In-depth interviews were conducted with 50 respondents: 23 community members, 13 MPs, and 14 policymakers and implementers. Among the 23 interviewed community members, 17 reported agricultural or livestock farming as their main occupation, two houseworkers, two general laborers, and two government officials. Most respondents were male (17 of 23), particularly community members because they were more likely to engage in farming and forest work that puts them at greater risk of malaria compared to their female counterparts. Apart from their main occupation, many also reported part-time employment in other sectors, such as construction work outside the peak agricultural seasons.

Two focus group discussions were conducted in Ubon Ratchathani and Sisaket provinces, with five and six participants respectively. The groups of five consisted primarily of elderly respondents, with two males and three females aged between 60–70, who have frequently visited the forest and experienced malaria. The other group of six consisted of four male and two female working adults who reported being familiar with the services the MP in their community provided.

Among policymakers and implementers within the health sector, five worked within the central government, three in sub-national government, two in international organisations, and four in civil society organisations. Eight were the directors or managers of their programmes, four programme officers, and two technical advisors or specialists. **Table 1** summarises the demographics of interviewed respondents. Detailed demographics and work profiles of interviewed MPs are reported in Table 3.

**Table 1:** Demographics of interview respondents (n = 50).

**Demographic information**	**Types of interview respondents**			**Total (% out of 50)**
	Community members	Malaria post workers	Policymakers/ implementers	
	23	13	14	50 (100)
**Sex**				
Male	17	8	9	34 (67)
Female	6	5	5	17 (33)
**Age range**				
20–29	1	0	0	1 (2)
30–39	4	2	3	9 (18)
40–49	7	6	5	18 (36)
50–59	4	2	4	10 (20)
60–69	6	2	2	10 (20)
70+	1	1	0	2 (4)

Initial stakeholder mapping results and key features of the current community-based malaria programmes from the rapid review of policy documents and reports [7, 16, 17], and characteristics of MP according to the DVBD’s RAI3E implementation plan are outlined in **S2 Appendix**.

The results from the interviews and group discussions are reported in the three sections below, focused on (1) the overview of community-based and facility-based health care; (2) potential roles and services that MPs could undertake in the future; and (3) challenges and opportunities related to the design and implementation of expanded services, considering the national health policy framework and the malaria elimination strategy.

### Overview of community-based and facility-based health care

The national malaria programme in Thailand is a semi-vertical programme under the national DVBD, implemented through a regional unit (OPDC) in coordination with the provincial unit (PHOs) under the general public health system. Among the key priorities, the National Malaria Elimination Strategy (2017–2026) endorses further public health system integration of community and facility-based health workers (VHV, MP, MC) as well as the health HPHs which are public health facilities providing primary care services to the populations in respective subdistricts, alongside building an effective surveillance system and expansion of services to cover the at-risk population. At the same time, Thailand’s strategy to decentralize its primary health care has been progressing through funding and task transfer to local administrative organizations (LAO) at provincial and sub-district levels across the country. **Table 2** depicts the multiple layers of government units under the vertical and general health systems involved in the development of community-based health programmes.

**Table 2:** Mapping of stakeholders involved in the management and implementation of community health workers in Thailand. The key documents reviewed were: 2011 WHO’s NMCP review report; 2014 MoPH’s primary health care division report; 2021 DVBD’s RAI3E Implementation Plan; additional web searches on government websites were conducted.

Domestic stakeholders		International stakeholders
National malaria elimination committee: Ministry of Public Health and other ministries, associations, academics, non-government organizations, and international organizations		
Department of Disease Control Division of Vector-Borne Disease	Department of Health Services Division of Primary Health Care	Donors: GFATM, BMGF Technical support: WHO, USAID Alight and the malaria CSO platform
**Local implementers**		
**Vertical malaria programme:** Office of Disease Prevention and control (ODPC) Vector Borne Disease Center (VBDC) Vector Borne Disease Unit (VBDU) Malaria Clinic (MC)	**General Health system:** Provincial Health Office (PHO) District Health Office (DHO) Community (district) Hospital Health Promotion (sub-district) Hospital (HPH)	**Civil society organization (CSO):** International Rescue Committee Raks Thai Foundation Shoklo Malaria Research Unit Stella Maris Young Muslim Association of Thailand
Malaria post (MPs) workers; Village health volunteers (VHVs)*; CSO volunteers		
**Community and population at risk of malaria**		

GFATM = Global Fund to Fight AIDS, Tuberculosis, and Malaria; BMFG = Bill & Melinda Gates Foundation; WHO = World Health Organization; USAID = United States Agency for International Development.

#### Profiles of community (health) workers

With years of residency and experience at MPs or in other community work, the workers were deeply embedded within their communities and the volunteer networks. All thirteen interviewed MPs had a minimum of seven years’ experience and were aged between 33 and 76 years. Ten undertook the role of VHVs under the public health system; four reported being the heads of VHVs supervising 8–15 other VHVs in their communities and many performed other roles such as village leader or village committee member, in the community, local administrative office, municipality, non-MOPH units, or a local civil society organization (CSO) (see **Table 3**). Their roles were primarily on a paid and part-time basis, without full time employment or official government status.

**Table 3:** Demographics and profiles of MPs in the study sites (*M = Male; F = Female*).

MPs	Sex	Age *(years)*	Years as MP	Education level	Monthly income in Thai baht *(USD)*	Previous and current experience in community (health) workers
MP1	M	55	14	Upper- Secondary	10,001–15,000 (331–500)	Recruited from VHV; resigned 5 years ago; active as assistant to the community leader
MP2	F	62	14	Upper- Secondary	3,001–5,0000 (101–150)	Recruited from VHV and active as head of VHV; active as non-MOPH volunteer
MP3	M	43	14	Upper- Secondary	5,001–10,000 (151–330)	Recruited from VHV and active as head of VHV; active as local representative
MP4	F	48	14	Upper- Secondary	3,001–5,000 (101–150)	Recruited from VHV and active as head of VHV
MP5	M	46	13	Upper- Secondary	5,001–10,000 (151–330)	Recruited from previous drug abuse program; active in the emergency services
MP6	F	33	11	Upper- Secondary	5,001–10,000 (151–330)	Recruited from VHV’s recommendation
MP7	M	76	12	Primary	3,001–5,0000 (101–150)	Recruited from VHV; resigned (did not specify years)
MP8	M	39	14	Lower- Secondary	3,001–5,000 (101–150)	Recruited from HPH’s recommendation
MP9	M	64	8	Primary	3,001–5,000 (101–150)	Recruited from VHV; resigned 2 years ago; active as community’s security volunteer
MP10	M	47	8	College	30,001–50,000 (1,001–1,500)	Recruited from VHV; resigned 3 years ago; active as member of the village committee
MP11	F	57	8	Upper- Secondary	30,001–50,000 (1,001–1,500)	Recruited from VHV; resigned (did not specify years)
MP12	F	44	7	Upper- Secondary	10,001–15,000 (331–500)	Recruited from VHV; resigned 5 years ago; currently active as CSO volunteer
MP13	M	62	10	Primary	10,001–15,000 (331–500)	Recruited from VHV; resigned 5 years ago; previously village health correspondent

#### Health seeking behaviours

Proximity to health providers emerged as one important factor affecting experiences of healthcare and access to primary care services in remote and resource-limited communities. In addition, providers of choice were often determined by the availability of resources, costs, and quality of care perceived and experienced by the communities. For example, even though malaria services at MCs and public hospitals were free of charge for Thai citizens, many respondents described excessive transport and time costs. Emergency ambulance (also referred to as the 1669 hotline) was also an option for emergency transport to the nearest hospitals for which MPs and/or VHVs in the same communities were a contact person. Having a transport service was highlighted as essential not only for malaria patients, but for the elderly and those without private vehicles.

Respondents reported varied healthcare costs for their households: this depended on their choice of health providers and the costs associated with each visit. Many reported receiving the free-of-charge health-related services from the volunteers and public health providers under the universal healthcare provision. More generally, HPH or health centers and district hospitals were visited for intensive medical procedures, surgeries, and other health conditions under the scheme. Visits to provincial hospital were usually reserved for necessary occasions, which may result in additional expenses from hospitalization or medical treatment. One respondent with health benefits from civil service was not concerned about medical costs as they were mostly covered during hospitalization or for advanced treatment.

*“I rarely visit a health facility but if I do visit a district hospital I pay around five hundred baht for transport, or a thousand (Thai) Baht to the provincial hospital. […] I use the golden card (referring to the universal health care scheme) so I do not need to pay for services there. My mother is also covered with the civil servant benefits. I like the health coverage. […] it should stay, so should the testing by MPs.”* Interview with female community member

Private clinics were reported as a provider of choice among adults in the communities for when their children were sick and required immediate care. Clinic visits were also common by those visiting the forest for treatment of scrub typhus with antibiotics prescribed by doctors or pharmacists at the clinics. Half of the respondents reported that they opted for private clinics after public operational hours or a public health facility on their days off work. Payment for service was acceptable for fast service and flexible opening hours, and occasionally for the quality of treatment they received compared to the hospital. In addition, provision of pain management injections, muscle relaxants, or medication for gastritis were perceived to be a more convenient treatment received from private clinics and pharmacies, especially among working adults and the elderly who participated in long and intensive agricultural activities during the day, despite having paid more than 500 baht for treatment each visit.

Three respondents mentioned that they were willing to pay for volunteers’ services if the fee was comparable to the same service from alternatives such as private clinics, or for transport to the hospitals. If they were to be charged for the testing service, however, one male respondent explained that he would not choose to be tested if he did not have symptoms.

#### Access to malaria services

For MPs, malaria testing with RDTs, treatment with antimalarials, consultation about adherence, and follow-up visits were perceived as their key and most valued roles in their communities. MPs reported that they were currently providing testing as passive case detection: either they were visited at the post (their own house), visited forest goers at home, at a farmhouse or routes they frequented. Due to significant decline in cases, some reported that they were recently tasked with targetting and testing 10–20 at-risk forest goers per month. All MPs reported that they were willing to continue their roles; often stating that this testing service should remain accessible given the distance from their village to the district hospital and road conditions.

*“Before the pandemic I would often go to see those at risk and offer testing…mostly near rubber farms where those at high risk work. […] These people work at night in the farms and sleep during the day. They would come down to sell their products and that was where I waited. […] If I did the testing randomly in the village, they would not come, you know, they would be resting at home. […] After the patients were treated we needed to follow-up with them on their treatment. I’d call to check whether they are taking the pills. (Interviewer: Why?) Because they may forget to take the medicines. They may not get fully treated and the disease may be resistant to the drugs.”* Interview with male MP

The proximity of MPs prompted at-risk groups to visit them: visiting MPs at their post was described as convenient for those who undertook long trips into the forest. In addition, they were popular with community members with a history of substance abuse, and young adults. Some shared their experience when they were reluctant to visits health facilities (due to fears of disclosing personal information) and perceived MPs as local residents rather than public officials.

*“I think those who visit us (MPs) have trust in us. They might be concerned about other tests that could be done at the hospital so when they are sick they may not go see a doctor. […] those who visit the forest are more likely to use illegal drugs. So they do not want to do any blood tests at a facility. They only come for a malaria test and nothing else. When malaria was very prevalent, […] I had 50–60 visitors in a month, sometimes almost a hundred. Back then, these people came to see me. Some people who went to the district got arrested so others were afraid to visit a public facility. Young people obviously are scared, some grown-ups too. This place is close to their homes. If they get malaria they know they get treated right away.”* Interview with male MP

Past clients described MPs as a convenient choice for prompt testing and treatment: this practice was also supported by their perception of malaria as a treatable disease and less severe than in the past when the disease was diagnosed and the patients sought treatment in time. Those aware of malaria and their risk preferred to get tested directly for malaria without having to spend up to half a day at the hospital, unless they had severe symptoms. Referral from MPs was also perceived as beneficial because RDT-positive cases were able to bypass queues to receive treatment at the hospital. All community members, with and without experience of malaria, preferred to receive mosquito repellents and hammock nets, citing these as necessary for their livelihoods.

Oftentimes, seasonal work and agricultural livelihoods deterred villagers from spending time away from work, especially those working in rubber plantations at nighttime and in early mornings. The main barrier to access hospital services for people in the community was the operational hours (9.00–12.00hr and 13.00–16.00hr Mondays to Fridays) during which many community members, especially those at risk of malaria, may be working in the farm or foraging in the forest.

Although those with experience of malaria described malaria testing byh MP as their preferred option, two respondents (one non-Thai and one Thai) reported disliking the services provided by the MP in their village. One described taking her child to the MP but being rejected for testing. Another reported disliking the MP’s ill manners, questioned her eligibility to treat fellow villagers, and suggested that the MP should treat the patients more equally or a new person should be recruited.

*“I wouldn’t visit the MP in my village. […] others might do. She did not get a degree. […] only trained to provide medicines. I personally do not know her and am not close to her. Even some of her cousins do not visit her. […] if she’s able to provide another service I still would not go. We did not argue but I do not like her personality. I feel more comfortable visiting to the hospital. […] it’s easier to ask my neighbor to take me there rather than visiting the MP.”* Interview with male community member

In one village, two respondents highlighted that MPs taking on multiple health (MP and VHV) and non-health (political or administrative) roles as having positive and negative reactions from their community, especially if the roles involve administrative responsibility or are likely to be politicized. While a one-stop service type of community-based worker was convenient, the village leader raised a concern about those who may not come for malaria services if they disapprove of the additional roles the MP undertakes. Suggestions were made that MPs and VHVs should work together to maintain access to care for all and to keep the activities transparent and trustworthy.

*“I think the MP in the village almost has too many responsibilities. She is wearing many hats (idiom for doing multiple roles). […] I would want other VHVs to help the MP with malaria activities. Working together also makes the activities more transparent. Also, because VHVs are already looking after households under their coverage, they can help distribute mosquito nets…better than only having only one person do all the work.”* Interview with male community member

### Potential roles and services of malaria post workers

#### Care for dengue, diabetes and other common illnesses

When we asked MPs about potential services that would benefit their clients, they most frequently mentioned dengue and diabetes. Dengue screening services were perceived as useful and preferable if the procedure was similar to that for malaria. At the time of the study, there were no testing services for dengue available in the health centers, although verbal and physical examination, for example a tourniquet test, may be performed. Some MPs suggested that they could offer dengue testing services since they had already routinely performed dengue foci investigation and management, namely observing and reporting household conditions to compile the house index (HI) and container index (CI) weekly reports for dengue surveillance. As one respondent explained, the provision of dengue testing would respond to a health care need in the communities and could facilitate the referral process.

*“There was a villager who once came to me for a dengue test but I told him that I only do malaria tests. So I told him to go to the health center. He wanted the test but I do not know about dengue, I only know about malaria.”* Interview with female MP

In addition, because their clients frequently visited the forest or forest farm, one MP suggested that services related to scrub typhus, information provision about how to prevent insect bites or symptoms, and treatment for skin inflammation and itchiness from insect bites, could be added. Blood chemistry screening was also specifically proposed by a local health worker for forest goers involved in “grey jobs” or illegal activities in the forest, such as logging and hunting, and potentially those who were heavily exposed to agricultural chemicals.

Care for diabetes was in high demand in the communities, especially among the elderly. One female MP requested training to do blood glucose testing for her patients, explaining that this service at the hospital usually took a long time. In addition, two MPs perceived case referral in general as important for many community members. The MPs and/or VHVs were commonly contacted to facilitate ambulance transport services, especially in an emergency situation or out of hospital working hours. Services related to patient referral, household visits and a general health check-up consultation or activities, such as measuring blood pressure for those at risk of high blood pressure, were suggested by MPs and some community members. Although these services were highlighted for the elderly, MPs perceived that the service would also be beneficial to those at risk of malaria who avoided physical examination at a public health facility.

To further align with primary care and HPH’s task as first point of care, suggestions from HPH staff were made that MPs could provide basic health advice about care seeking and information for common illnesses, such as diarrhea or fever, and illnesses or health issues that benefited from initial consultation and referral support, such as administering screening surveys on health conditions for mental illnesses or substance abuse. Substance use was also recognized as related to mental illnesses in the border areas; additional services in these communities could be provided to recovering drug users post-therapy for monitoring and follow up. These two tasks were, however, perceived as complicated, requiring the patient’s consent or support, and raising safety concerns for the workers themselves.

#### Emphasis on screening (rather than diagnosis)

Policymakers and implementers suggested that MPs could take on the role of screening for common illnesses beyond malaria, including for other infectious diseases, and non-communicable diseases. In addition to authorised testing by health volunteers, which includes glucose and malaria testing, several respondents suggested using RDTs for other health conditions such as COVID-19 or dengue. However, it was emphasised that the patients screened by volunteers should be referred and properly “diagnosed” by a medical professional, and that an invasive procedure, such as performing a rapid test using blood samples, should be under close supervision of an authorised professional.

*“We have consulted with the provincial health office and they seemed to agree (to train volunteers to do RDTs) but we also need to advocate for this at the policy level. There are many conditions […] but we have concerns about blood testing. From our current project under this grant (referring to the RAI3E), we received support from the province in Kanchanaburi, to train VHVs to use malaria RDTs. In Sisaket we found that there were not enough RDTs for VHVs to use even though we are allowed to train them. So we focused on building communication skills and this was well received by the volunteers. These tasks, tasks they were assigned to, made them proud. […] prouder than being a regular VHV really because they were capable of doing so much more.”* Interview with CSO implementer

With various suggestions regarding potential tasks, either to have MPs provide new testing services beyond malaria or have VHVs perform additional malaria testing, there was emphasis on the legal barriers to volunteers performing testing and taking blood samples, although an exception has been made for malaria testing by MPs. Taking blood samples and testing with RDTs were described as special tasks that could only be performed by MPs who received regular training by the respective disease departments and under the supervision of eligible medical staff, currently the provincial chief medical officer. Many respondents perceived the use of RDTs for malaria and non-malarial diseases as feasible if performed under close supervision of public health staff. One suggestion was that MPs could strengthen the services they themselves already performed as VHVs, such as prevention and surveillance of dengue, by adding verbal investigation or health education sessions for their clients. Administering a test for dengue was, however, seen by some respondents as more complicated than malaria RDTs which requires specific training, including proper case investigation, reading of results, and close supervision of the volunteers by medical professionals.

#### Overburdened, underpaid, and underperforming

While there was agreement about the potential public health benefits of expanded services, three primary concerns emerged. Many MPs, particularly senior workers, reflected on their large workload from testing the at-risk population and following up malaria patients, which was as high as a hundred patients per month during the 2014–2017 outbreaks. Many described the large volume of patients and the fact they were able to help them recover as their main motivations for becoming an MP. Some, however, complained that the compensation they received was not sufficient, especially in the past when MPs had to conduct a follow-up visit of patients at home or in the forest. One MP perceived the compensation he currently received while cases were declining as substitution for his past out-of-pocket expenses from following up many cases on a monthly basis. Many felt overburdened by their additional roles as VHVs during the COVID-19 pandemic, and some complained that the renumeration was not proportionate to the time spent and risk of self-infection.

*“I conducted active case detection every year. In the past there were a lot of villagers coming to take the tests. But this year I said I would prefer not to go. […] I called my supervisor and told them that I’d be at high risk. […] we’re just volunteers, you know? What if I get infected (with COVID-19), what would I do? In the end, the province had MPs vaccinated as well (like other healthcare workers) so we could continue to work.”* Interview with female MP

Most MPs described their current tasks as manageable: using RDTs, making slides, and administering medicine did not exceed their capability. Senior MPs, however, reported being unable to effectively use their mobile phones to perform some tasks, such as online case reporting, and were concerned that this would affect their performance in their future roles. Younger MPs reported that using smartphones has improved their work, especially by facilitating their communication with their clients and public health officials during the pandemic. They were, however, concerned that limited internet connectivity in the forested villages affected their regular communication, and that the cost of utilising the technologies was borne by themselves.

Training was crucial for MPs to be accepted by their clients, and MPs felt more confident if the training was conducted annually. Beyond training and supervision, MPs appreciated collaboration with other local health providers and programme staff to implement malaria interventions, including health education, mobile malaria clinics, mosquito net distribution, and mass testing, and were keen to provide continued support despite the decline in malaria. One MP suggested that communal sensitisation meetings, which often accompanied these interventions, were a suitable platform to inform communities about the MP’s current services or other potential roles in the future. Three MPs reported that they had already been invited to participate in health programmes related to HIV and non-communicable diseases (NCDs) in their communities, either organized by public hospitals or non-governmental organizations, while performing their MP roles. And this kept them informed and able to refer their patients to these specific programmes when needed.

*“There are NGOs that work on malaria and HIV programs in these areas. (Are there many HIV patients in your villages?) Not in my village, but in other two villages nearby. The staff have done a lot of screening of villagers, especially for the migrant population, but they were not able to provide malaria testing or treatment. For HIV, my role is to suggest to villagers that they should get tested, especially those who recently moved back to the village.”* Interview with male MP

The MPs identified specific support needs to perform additional roles. Although compensation was not cited as their primary source of motivation, MPs suggested additional financial incentives, such as fuel cost for motorbikes or mobile data plans, should be assigned for household visits, patient follow-ups, or online real-time case reporting. Although MPs suggested they were already performing extended roles as VHVs and working together with the volunteers, to minimize overlapping tasks with VHVs in their communities, they requested that the additional roles be complementary or upgraded from the existing tasks, to avoid being overburdened.

*“We have tried to introduce more incentives for volunteers. During the (COVID-19) pandemic, for example, we advocated for compensation for occupational risk to encourage and provide moral support to the VHVs. […] At the time they seemed to be overloaded with the pandemic-related work. Another example: we know the Caregiver programme that trained caregivers to provide care for the elderly (disabled person and bedridden patients). This work was previously assigned to VHVs (before new caregivers were recruited and trained) and this programme helped remove the burden from the volunteers. These are the challenges VHVs have raised and that we tried to respond to.”* Interview with national policymaker

### Prospects about future programme design, implementation and policy considerations

Two key considerations emerged when discussing prospects around further development of the MP programme: (1) the need for alignment with the national guidelines on health volunteers and local priorities, and (2) its contribution to progress towards overall malaria elimination.

### Alignment of volunteer roles with national guidelines and local health priorities

Many respondents (government and non-government) addressed questions about expanding MPs’ roles with an acknowledgement that MPs may have already performed other services beyond malaria–as VHVs, or through other volunteer work. Although the malaria programme has remained “semi-vertical”, progress towards health system integration was illustrated by the management of MPs by PHOs under the general health system. A DVBD respondent proposed to reframe the question in terms of “integration” rather than “expansion”, and highlighted that any plans for programme development should be in line with the ministerial regulations regarding VHVs, especially the tasks required to be under the supervision of medical professionals or technicians. A participant from a CSO argued that further development of MPs should consider findings from an evaluation of their performance of malaria (and non-malaria) services. An international implementer also suggested that any updates of community-based health services, including those provided by malaria posts, should respond to epidemiological changes and local demands (Box 1).

*“So the point is […] that we need to have better tools to be able to do this kind of assessment […] like participatory rural appraisal […] you are more of an observer rather than someone coming in with preconceived ideas. You try to understand what’s really going on and then design something that is applicable for that context. […] If you want to have an impact, especially now in elimination with declining burden, there are so many other priorities in these communities even at the border […] malaria is not the biggest problem. The people are affected by so many other different things like employment, fleeing from conflict, being separated from families and all kinds of mental health problems. So there’s a huge health concern and it’s not just malaria. And if we address it only as malaria we will always struggle, I think, to find the right approach.”* Interview with international organisation implementer

**Box 1. Current tasks, suggested tasks, and examples of successful cases in the adoption of point-of-care testing by trained health volunteers; extracted and summarized from the 2011 public health ministerial regulations regarding village health volunteers (updated version 2013) and interviews.**

**Table:** 

Tasks in the current terms of references, according to the ministerial regulations • Basic care to alleviate symptoms (such as fever, diarrhoea, pain/ache, anaemia, skin rashes) • First aid (such as for wounds, burns, animal bites) • Blood testing for malaria • Provision of household common drugs, herbal infusions, or medicine • Glucose test Suggested new roles and tasks • Screening for symptoms of non-malaria diseases and case referral (such as for unspecified fever) • Screening using other rapid test kits (such as for diabetes, dengue, blood biochemistry) • Providing basic care for non-malaria illnesses (such as measuring blood pressure, providing Oral rehydration salts for diarrhoea, giving first aid care) • Health information provision for local health priorities (such as diabetes) and communicable diseases (such as dengue, COVID-19) • Providing basic consultation and referral (such as for mental illness or substance use pre- or post-treatment)

#### Progress towards elimination, horizontal integration, and challenges

Interviewees from the DVBD highlighted how, as malaria elimination progresses, they are prioritizing horizontal integration, with responsibility for malaria programme implementation transferred to the general health system. Respondents observed that this strategy will initially focus on low transmission areas and then it will be extended to endemic areas, where vertical malaria services are still needed. However, policymakers also highlighted barriers to horizontal integration due to resistance to change and human resource challenges:

*“The malaria programme in Thailand is still semi vertical…we have planned for the integration of malaria services into general public health and we are still at the beginning of the integration, a gradual integration […] starting from provinces that are near elimination first. But in the area that still sees high prevalence of malaria, we still keep the vertical programme. […] Our priority is to extend the programme in high endemic villages…to have MPs funded by the local sub-district administrative units […] We need to speed up the integration because many of our staff (under the vertical programme) will retire soon. But this is not easy and we may face resistance from both the vertical programme and the general public health system. So what we are trying to do is to strengthen the capacity of local authorities to invest in malaria activities in their areas. At the same time, we trained staff at health promoting hospitals to test and treat for malaria.”* Interview with national policymaker

Most respondents agreed that the public health system is well equipped to perform malaria case management and surveillance, but highlighted that multi-sectoral coordination with NGOs and border care services is still needed to control cross-border malaria and reach elimination. At the time of the interviews, implementers raised concerns regarding the challenge of eliminating *Plasmodium vivax* malaria along the Thailand-Myanmar border. There were lesser concerns about elimination challenges in the other regions, including those bordering Lao PDR and Cambodia, where the movement of mobile and migrant populations was minimal. However, many agreed that community-based malaria services remained essential in areas where at-risk groups may still travel to malaria hotspots after the COVID-19 travel restriction had been lifted, such as in Ubon Ratchathani and Sisaket.

Although these workers were perceived as essential for malaria elimination, questions regarding cost-efficiency for the national malaria programme were raised if MPs in low-transmission areas were to be maintained. Suggestions were made to increase the contribution of MPs to the country’s elimination goals including surveillance and prevention of re-establishment, for example, performing active case finding, treatment adherence consultation and monitoring, and supporting the 1-3-7 strategy for all reported cases in their communities. Implementers noted that the quality of surveillance data and the adoption of mobile technologies were being increasingly prioritized to improve the malaria information system (MIS) and integrate this with the Health Data Center (HDC) dashboard under the primary health information system. This could also be a role that MPs in near-elimination provinces can fulfill to maintain zero cases for three consecutive years to be certified malaria free.

There was some consensus that, in the long run, these upgrades could benefit from the ongoing process of health sector decentralization and the increasing availability of local funding pools. Both national and local implementers agreed that local stakeholders are more in touch with the communities and are more aware of local priorities, compared to central-level managers in the malaria programme; thus, their roles and function should be defined by local stakeholders.

*“In this transition process, we work with DVBD to involve other partners like the hospitals and community health workers, and train these people and develop the guidelines for the partners. We should empower the local partners…with the decentralization, the primary care units should be stronger. …the trained health volunteer is the person closest to many of the population … I think the government should make it clear, under this act, that these community health workers could have more capacity. I used to work at a refugee camp, where there were no doctors. The health workers need to understand the serious side effects and should be able to treat at least with paracetamol, for example.”* Interview with international organisation implementer

Decentralisation of funding was also perceived as a favourable development which may provide the needed resources to support local malaria activities. However, both national and subnational policymakers foresaw that a funding proposal for only malaria activities would face competing priorities with high burden diseases such as dengue and diabetes.

## Discussion

This study drew on interviews with fifty stakeholders, including policymakers, implementers, healthcare service providers, and community members living in malaria endemic communities, to explore the prospects of expanding the roles of MPs beyond malaria. Whilst all respondents were able to identify potential roles and functions of MPs, many also pointed out that these roles should be aligned with achieving the overall integration strategy of the malaria programme, which is to embed malaria activities with the local health network and primary care system.

The decline in malaria cases and scarcity of resources have driven the efforts to integrate the national malaria programmes in Thailand [18] and other GMS countries [19, 20]. Integration of these programmes into more comprehensive community-based programmes, such as integrated community case management (iCCM) [21] and wider programmes on vector-borne diseases [22], was seen as a solution to ensuring the continued provision of malaria services and surveillance as well as essential care for other public health priorities in the communities. In addition, community health workers can be trained to address public health emergencies. Recently, as the communities were locked down during the COVID-19 pandemic, multiple groups of community health workers, including VMWs, were leveraged to provide additional health services to support COVID-19 prevention and control [23, 24]. Based on the success of these interventions, Thailand’s health policymakers may be interested in expanding the roles of MPs to address other important health needs and priorities in the communities, including NCDs.

The perceived health concerns identified in our study were reflected in 2022 health programme reports from the provincial health departments: diabetes, hypertension, musculoskeletal disorders, gastrointestinal diseases, and mental illnesses, for example, contributed the highest burdens of diseases in Ubon Ratchathani [25] and Sisaket [26]. In addition, acute diarrhoea, pneumonia, hand, foot and mouth disease, melioidosis, mushroom poisoning, and food poisoning were also identified as priorities for disease surveillance in 2022. Health providers and subnational policymakers in our study suggested that volunteers such as MPs could be trained and equipped to address these health priorities in the communities.

In Southeast Asia, COVID-19 disrupted the provision of essential services for other infectious diseases and NCDs [27], with potential long-term health impacts. Addressing these and future health priorities is thus essential to ensure continued relevance of community-based programmes. As some participants suggested, further evaluations, such as community needs assessment [28] or participatory rural appraisals (PRA) [29], should be conducted to gain a better understanding of these changing priorities and develop community-directed interventions [30].

The increasing availability of novel low-cost diagnostic tools for community-based testing has generated further interest in the potential expansion of vertical programmes [31–33]. In addition, new mobile applications can transform smartphones into readers of rapid tests, assisting users with basic diagnostic skills and thus increasing accuracy of community-based testing [34]. These innovations have great potential to strengthen community-based care in rural areas, especially where access to health facilities is still limited. Implementers, healthcare providers, and communities were interested in the potential of testing kits for diabetes, dengue, and COVID-19, and data reporting or screening tools using smartphones which could contribute to accelerating the screening and referral process in the primary care system. However, it was recognized that the selection of volunteers performing testing services would need to be reviewed for their eligibility, and training and supervision provided according to national guidelines.

At the policy level, key requirements to support the development of expanded programmes included the need for political collaboration and financial support. An investment case, advocating more domestic funding has already been made [35], as well as promoting further engagement of local administrations to fully leverage their capacity and financial resources [36]. The need for support from local health networks (including provincial and district health offices, community hospitals, local administrative offices, HPHs, VHVs), communities and civil society to sustain MPs and integrate their services with the primary care system was raised by policymakers and implementers, who saw the opportunity to build on the presence and skills of MPs and strengthen their roles, especially for the referral of non-malaria cases to public health facilities. Perhaps lessons could be drawn from the dengue control programme, which was successfully implemented in collaboration with the local administrations [37]. The ability to provide more comprehensive services at the community level, not only for malaria, but febrile illnesses more broadly, has been identified as crucial to the reinforced primary care system, especially in reaching populations at the periphery.

Helping other community members has been identified as a core value of community-based work in previous studies [38] and was the primary motivation for many volunteers in this study. This disposition should be further encouraged, supported and recognised in terms of social standing and rewards. Local volunteers are entrepreneurial and often must undertake multiple jobs to maintain their livelihoods. There is much potential to empower these volunteers to become a stronger link between remote communities and the health system. Their roles as gatekeeper and community representative were highlighted in leveraging collaboration of villagers, political leaders, and public health officials during the pandemic [39–41]. However, the limited digital literacy of many senior workers could constrain their ability to utilize smartphones and perform technology-based roles if assigned without capacity-building or supervisory support. Programmes aiming to expand or integrate the roles of MPs or any other volunteers should focus on addressing other programmatic and contextual factors such as the current roles of volunteers in the communities, the support of local health networks, and the prioritized health programmes [42] that enable their performance and ensure successful programme extension.

**Box 2** contains the recommended actions and considerations for programmes aiming to expand the roles of MPs in. Recent evidence on outcomes of expanding the roles of VMWs could be further explored earlier in Myanmar and along Thailand’s western border [43, 44], as well as feasibility studies in Cambodia [45, 46] and a stakeholder analysis in Laos [47].

**Box 2. Considerations for MP role expansion and/or integration strategy.**

**Table:** 

1. Potential roles should be based on primary care essential services guidelines as indicated in the following: screening symptoms; health information provision; basic care ‐ diarrhoea, first-aid; diabetes & blood pressure screening. The list should also be adaptable to changing local needs, such as documented in Ubon Ratchathani and Sisaket, including local health concerns for diabetes, cancer, malaria, febrile illnesses, dengue, and diarrhoea. This could be supplemented by results from rapid health assessment using approaches such as participatory rural appraisal. 2. Linkage between the MPs and the wider local health system should be strengthened especially in support of their roles in screening programmes and referral to the public health facility while considering the capacity of local health networks to support MPs and VHVs as the referral link. 3. Legal constraints for their testing role should be clarified ‐ making a clear distinction between “testing” by volunteers and “diagnosis” by medical professionals; requirements including certification, training, performance evaluation, and supervision should be designed and planned by programmes and implementers. 4. The role of local health network involved including MPs’ supervisors should be strengthened within the provincial health offices, public hospitals and civil society organizations through multisectoral coordination, to support MPs and increase awareness of the communities 5. The undertaking of multiple volunteer roles of MPs, especially those who are also VHVs, should be documented if priority will be given to those who are already VHVs until certified malaria free–integrated MPs and/or VHVs are likely to receive further funding by the local authorities and health networks. This is to be further supported by a clear direction for integration among MOPH, CSOs, and local health network in the long run 6. Capacity building for MPs and VHVs to integrate data collected within the general public health information systems and the malaria information system should be planned and piloted to support malaria surveillance interventions in low transmission settings. 7. The national malaria programme should continue to monitor and evaluate malaria activities as well as any expanded roles and activities performed by MPs.

### Strengths and limitations

Although in-depth interviews with central-level policy makers and health sector managers were conducted, findings from in-depth interviews with implementers and community members in two provinces cannot be generalized nationwide. Perceived illness experiences and health concerns, although extremely valuable, may not reflect the true burden of diseases in the areas. This was supplemented with secondary data from the provincial levels (burden of diseases, and prioritized disease surveillance in 2022), and more research conducted in the region. Although attempts have been made to recruit respondents from diverse groups, we did not interview former MP workers who chose to resign or were withdrawn from the programme due to relocation strategy, and policymakers outside of the Ministry of Public Health who may offer more insights on the wider policy framework, such as the Ministry of Finance or Local Administration Office, for the future development of community-based health programmes.

Drawing on the perspectives of stakeholders at multiple levels, this study contributes to a comprehensive understanding of community-based malaria care in Thailand. The intention was to document the expectations of these roles, as well as explore the complex nature of the workers’ set-up, complementing earlier studies that described their specific roles in malaria interventions [4, 6, 10, 48]. However, it might not fully investigate their other roles beyond malaria programmes. The qualitative methods and iterative approaches enabled the prospective policy analysis, supplemented by the policy document and desk reviews, to inform the design and implementation of malaria programmes in Thailand, and countries the GMS considering a similar strategy.

## Conclusion

The findings suggest that expanding and integrating the role of MPs is not without challenge. Any extended programme should be designed according to local needs–some of which have been identified in this study–recognising these needs are subject to change. It is also critical to look at the prospects of expanding their roles in communities where they often take on multiple, different and sometimes overlapping roles. Malaria is no longer perceived as a major health threat in the communities where immediate concerns are NCDs, mental health and wellbeing. To sustain community-based malaria services, it is essential to build on the capacity of the primary care system and local administration, and strengthen these valuable workforces via strengthening links between peripheral communities and the health system until elimination is reached.

## Supporting information

S1 Appendix In-depth interview and focus group discussion guides. (DOCX)

S2 Appendix Key features and implementers of community-based malaria programmes. (DOCX)

S1 Checklist Inclusivity in global research. (DOCX)
