# Supplementary material for: Expanding the roles of malaria post workers in Thailand: A qualitative study of stakeholder perspectives
Source: PLOS Glob Public Health. 2024 Sep 17;4(9):e0003670. doi: 10.1371/journal.pgph.0003670 (PMC11407649; doi:10.1371/journal.pgph.0003670)
Supplement: S1 Appendix — (DOCX) [file pgph.0003670.s001.docx]

**S1 Appendix. In-depth interview and focus group discussion guides**

**1.1 Interview guide for policy makers and implementers**

Aim of the interview: to identify and describe the perspectives of policy makers and implementers on expanding the roles of malaria post workers (MPs) and/or village health volunteers (VHVs) and community-based health programmes implementation.

**1. Introduction and background questions**

Thank you very much for participating in this interview. To begin with, could you tell me something about your background and current work?

**2. Health sector gaps**

As part of this project, we would like to gain a better understanding of challenges to health care delivery in remote rural communities. What are your views?

Probes:
Focus on the management of febrile illness, including diagnosis, referral, and treatment (with examples)

- Consider barriers in access to services across different dimensions: geographic accessibility, availability, affordability, and acceptability
- Encourage discussion of challenges associated with specific population groups (e.g. ethnic minorities, women) and health concerns

**3. Policy prospects**

What is the current policy framework to address these gaps? What are the plans for the future?

Probes:

- Focus on different stages in the policy cycle: prioritisation, policy formulation, and policy implementation
- Discuss potential policy challenges but also opportunities (such as upcoming rounds of donor funding)

**4. Expanding the role of village health workers**

[If not mentioned earlier]. Do you think MPs and/or VHVs could be used to address these challenges? If so, how, in what capacities?

Probes:

- Encourage retrospective analysis of previous experiences with village health workers, particularly for the management of malaria and TB
- Encourage a reflection on specific challenges to, and opportunities for, the expansion of VHWs, including policy and operational challenges
- Mention specific issues such as financing and sustainability, integration in the wider health sector, and motivation

**5. Feasibility of rapid diagnostic tests and micro-technologies for community-based interventions**

As part of this project, we envisage the introduction of low-cost, rapid tests which can be used to diagnose and manage non-malarial febrile illness and other diseases in the communities. For example, [SHOW PHOTO OR SLIDES]. What do you think? Do you think this could be a good idea? Can we discuss potential challenges and opportunities?

Probes:

- Direct the discussion around the key issues of sustainability, usability, procurement, acceptability, feasibility and integration into the wider health system
- Encourage a reflection on past experiences with rapid diagnostic tests for malaria

**6. Conclusions**

Thank you very much. Would you have anything do add? Do you have any questions? Would you have any suggestions about specific areas of investigation we should consider in future interviews? Could you name other informants we should talk to?

**1.2 Interview guide for malaria post workers**

Aim of the interview: to identify and describe the perspectives of MPs (and/or village health volunteers) on expanding their roles and support needed to implement the extended services.

1. **Background information**
   1. Place of residence: Where do you live? With who? How long have you lived in the village?
   2. Occupation: Are you currently working? What do you for a living?
   3. MP/VHV: For how long have you been a MP and/or VHV? How many years?
2. **Current tasks and experience working as MPs**
   1. Current role and service(s)

- Can you tell me about your role as a MP? What do you do?
- What service(s) do you provide?
- What kind of activities do you do?
- Where do you provide the service(s)?
- When do you provide the service(s)?
  1. Clients
- To whom do you provide the service(s)?
- Who comes to see you? How many? How often?
- When do they come to visit you?
- If they do not come to visit you, where do they go? To whom?
  1. Recruitment, training, and supervision
- How did you become a MP? Were you recruited? By who?
- How much are you paid for the role/task? By whom? How often?
- Do you receive any training for the job? How often?
- What is the training for? What is your opinion about it?
- Do you receive any supervision for the job? How often?
- What is the supervision for? What is your opinion about it?
  1. Main benefits and drawbacks or work challenges
- What do you think about your experience as a MP?
- What do you like about it?
- What do you dislike about it?
- Are there any benefits from your work? What are they?
- Are there any drawbacks/disadvantages from your work? What are they?
  1. Work challenges
- What do you think are the main challenges for you to perform your role?
- How do you cope with them?
- Are they any problems for you to complete your work? What are they?
- How do you fix them?
- Is there anything you think could be improved?
  1. Future plan and motivation
- Would you like to continue your MP role in the community? Why (not)?
- For how long do you think you will be a MP?
- If you could change anything about the role, what would it be?
- Where do you see yourself in 10-20 years?

1. **Perception of expanded roles and additional services beyond malaria**
   1. Expanded roles

- What do you think about expanding your role as a MP beyond malaria?
- What do you think could be the benefits for the expansion?
- What do you think could be the drawbacks for the expansion?
  1. Additional service(s)
- What additional service(s) do you think should be provided in your community? (Probe with list of additional services) (e.g. antenatal care, family planning, vaccination)
- For which disease do you think the service(s) should be provided? (e.g. tuberculosis, pneumonia, diarrhoea, malnutrition, HIV/AIDS, cancer, diabetes)
- What kind of activities do you think could be provided? (Probe with list of activities e.g. prevention, diagnosis, treatment, follow-up, referral, health education)
- What are the main health concerns among your community members?
- Are there specific groups the services should target?
  1. Feature of expanded role(s) and additional service(s)
- Would you like to provide those service(s)?
- How do you think those service(s) should be provided?
- Where do you think the service(s) should be provided?
- What are your preferred working hours?
- What do you think about payment for the service(s)?

1. **Necessary support to implement extended services**
   1. Types of support

- What support do you think is needed for you to implement these additional roles?
- What do you think you will need to provide the additional service(s)?
- What skills/training do you think are important for you? How often should they be?
- What supervision do you think is important for you? How often should they be?
- What tools/supplies do you think are important for you to have?
- How would you like to be compensated for the additional service(s)?
- Are there other types of support you need?
  1. Available health services in the community and partners
- Who are the other healthcare providers in your community?
- For which services do the community members visit the other providers?
- Do you work with those providers for any of your tasks/roles/services? On what aspects?
- With whom do you collaborate to perform your tasks?
- What collaboration would be beneficial for you to provide the additional service(s)?

**1.3 Interview guide for community members**

Aim of the interview: to identify and describe community members’ experiences with, and expectation of, health services provided by MPs and other health care providers.

1. **Background information**
   1. Place of residence: Where do you live? With who? How long have you lived in the village?
   2. Occupation: Are you currently working? What do you for a living?
   3. Health concern: Do you have any health concern? For what disease(s)? Do you have an illness? Do your family members have an illness?
2. **Experience with available healthcare services in the community**
   1. Awareness and uptake of health service providers

- Do you visit other health services provider? (e.g. traditional healer, health centre, hospital, clinic, pharmacy)
- For what reason/service do you visit the provider(s)?
- How often do you visit the provider(s)?
- What is your experience visiting the provider(s)?
- What do you like about them?
- What do you dislike about them?
- Where are these services? How far are they from your house?
- Do you have any problems accessing the service(s)?
  1. Cost
- How much do you pay for healthcare now? In a month? In a year?
- For what services do you pay? How much do they cost?
- How do you pay for the service(s)?
- Are there other costs from the service(s)? (e.g. transportation, work loss)
- Do you have/receive any health benefits scheme? Can you tell me about it?

1. **Current experiences of MP services**
   1. Awareness and uptake

- Do you know a MP in your community? Who is s/he? Where does s/he live?
- What services does the MP provide?
- How many MPs are there in your community? Do they provide different services?
- Have you ever visited the MP? For what reason/service?
- How often have you visited the MP? How many times?
- Has your family visited the MP? For what reason/service?
  1. Access
- How far is the MP from your place? How long do you take to get there?
- What time do you usually visit the MP?
- Are there times when the MP was not available?
- Do you have to pay for the services?
- Are there other costs from visiting the MP? (e.g. transportation)
  1. Satisfaction
- Are you satisfied with the service(s) provided? Why is that?
- What do you like about the services?
- What do you dislike about the services?
  1. Acceptability
- What is your experience with the MP?
- What is your experience with the services?
- Are you comfortable to receive the services from the MP?
- Do other people in the community visit the MP? For what reason/service?
- If not, who or where do you/they visit?

1. **Expectations on current and expanded MP services**
   1. Benefits/drawbacks

- Do you think the services benefit your community? In what way?
- Do you think the MP services are needed in your community? Why do you think so?
- Are there any drawbacks about the services?
  1. Quality of service
- What you think about the quality of the services you received?
- Do you feel better after you received the services?
- What could be improved?
  1. Preferences for other additional services and MP to pay
- What other health services do you think the MP can provide to your community? Probe with list of potential services
- What other health services do you think will be beneficial? Why do you think so?
- How much do you think you would pay for the additional services provided by the MP?

**1.4 Community focus group topic guide**

Aim of the focus group discussion (FGD): To explore local health concerns in the community and how the expanded malaria post workers (MPs) and/or village health volunteers (VHVs) roles could be developed and implemented to better reach specific groups of community members.

1. **Introduction and overview of activities** (approx. 5-10 mins depending on the group size)

- Welcome, introduction of staff and project, introduction of FGD participants
- Purpose of the FGD
- Ground rules

1. **Local health concerns and population-at-risk in the communities** (approx. 20 mins)

- What are your biggest health concerns for yourself?
- What are your biggest health concerns for your children?
- What disease(s) are you and/or the community concerned about?
- Who are the population at risk for this/these disease(s)? Who experiences it/them the most?
- Where do community members go to get treatment for the disease(s)?
- Which health services are already provided to the community?
- Are there difficulties accessing these services in your community?
- Do you have to pay for the services? How much?

*These questions can be discussed for a quite a long time, about 20 minutes, so that the participants can recall their experience and make comments.*

*At this point they may mention some “health problems”, “target population”, “health service providers” spontaneously 🡪 note-taker will note the ideas mentioned on the board/on a piece of paper, and discuss them later.*

- Can you think of anything else? Try to think of as many things as possible.
- What do you think about this problem/disease? *use list of possible health problems/diseases*

*If the lists of “health problems” is exhausted, we can ask about the concerns in more detail:*

- Which concerns/diseases do you think are the most important for your community? Please rank the concerns/diseases in terms of importance
- Does everyone agree to this ranking? What do you think should differ?

1. **Expectations on and preference for additional services provided by MPs** (approx. 20 mins)

- How is the malaria situation in your community?
- What do you think about the current services provided by MPs?
- How could they be improved?
- Do you have any complaints about the services? Did anyone else?
- Are there other health services in addition to malaria that could be added?

*🡪 Note down “health problems” and “target population” again*

*🡪 Add “health services” to the board/note.*

- Can you think of anything else? Try to think of as many things as possible
- What do you think about this service? *Use list of possible health services*

*If the list of “health services” is exhausted, we can ask about the services in more detail, e.g.*

- Which services do you think are the most important for your community? Please rank the services in terms of importance

E.g. Person A, you said you think “service X” is the most important. Can you tell us more about it? Why do you think so?

- Does other people agree with this ranking?
- What do you think should be different?

1. **Design and feature of implementation of expanded MP roles** (approx. 30 mins)

*Sum up the health concerns and additional health services you have noted down on the board/paper.*

*The aim is to discuss the most important additional health service in depth, or all the services if possible. Choose a specific service and ask about its implementation in more detail:*

*🡪 Note down “target population” again*

*🡪 Add “activities” to the board/note*

*🡪 Add “community”*

- For this service, what kind of activities do you think should be provided to the community?
- Is there a specific group the service should target or be provided to?
- How do you think the service should be provided? (e.g. home-based delivery, by MPs, referral)
- Would you be willing to receive this service from a MP?
- What do you think are the advantages if a MP providing this service?
- Are there any disadvantages? What are they?

*Community engagement and sustainability of the services:*

- Do you think the community could contribute to the service provision? How? In what way?
- For how long do you think the services should be provided?
- If you need to pay for the service, would you be willing to pay and how much?
- How do you think the services could be sustained in your community?

1. **Conclusion** (approx. 10 mins)

*Sum up what has been discussed, mention the positive aspects, and thank the participants.*

- How did you like talking about the topics with us?
- Is there anything important to you we have not mentioned?
- If you want to follow any issues you have talked about, you can contact us. (We will be at …. during this period … or contact us at …)
- The participants will be told that if they want to withdraw from the study, their data will not be used.
