# Supplementary material for: Expanding the roles of malaria post workers in Thailand: A qualitative study of stakeholder perspectives
Source: PLOS Glob Public Health. 2024 Sep 17;4(9):e0003670. doi: 10.1371/journal.pgph.0003670 (PMC11407649; doi:10.1371/journal.pgph.0003670)
Supplement: S2 Appendix — (DOCX) [file pgph.0003670.s002.docx]

**S2 Appendix. Key features and implementers of community-based malaria programmes**

**2.1 Overview of malaria and community-based malaria programmes.** Source: 2011 World Health Organization (WHO)’s Thailand National Malaria Control Programme’s review report; 2014 Ministry of Public Health’s primary health care division report; 2021 Division of Vector-Borne Diseases (DVBD)’s RAI3E Implementation Plan; additional web searches on government websites were conducted. Data from the interviews are used to supplemented the extracted information.

| **Key features** | **Implementers (stakeholders identified)** |
| --- | --- |
| - **Thailand’s national malaria control programme (NMCP)** is semi-vertical programme under Division of Vector-Borne Diseases (DVBD), Department of Disease Control (DDC), and implemented through Office of Disease Prevention and control (OPDC) in coordination with Provincial Health Offices (PHOs) under general public health system. - The malaria programme is primarily funded by the national government. The programme is also part of and receives funding from the Regional Artemisinin Initiative 3 Elimination (RAI3E). DVBD and the Global Fund management office under the DDC is Co-Principal recipient; DVBD is sub-recipient; ODPCs are sub-sub recipients of RAI3E grant. - There are 7 modules under RAI3E implementation plan:   (1) Vector Control which includes: entomological monitoring; support for Long-lasting Insecticidal Net (LLIN) and Long-lasting Insecticidal Hammock Net (LLIHN); Information Education Community (IEC)/Behavioural Change Community (BCC); and Indoor Residual Spraying (IRS)  (2) Case Management which includes: Active Case Detection (ACD) and Investigation; Facility Based Treatment; Integrated Community Case Management (ICCM); Private Sector Case Management; Therapeutic Efficacy Surveillance; and IEC/BCC-Case Management  (3) RSSH: Laboratory Systems which includes Information  Systems and Integrated Specimen Transport Networks; and Quality  Management Systems and Accreditation  (4) Community Systems Strengthening which includes Social mobilization, building community linkages and coordination; and Community Based Monitoring  (5) Specific Prevention Interventions which includes IEC/BCC  (6) RSSH: Health Management Information System and M&E which includes Program and Data Quality; and Routine Reporting  (7) Program Management which includes Grant Management.   - Malaria clinic (MC) staff were a mix of programme staff and government employees with expertise in microscopy diagnosis and vector control measure implementation; however many would soon be retired and the program would lose its workforce under its vertical programme. - According to 2016 National Malaria Elimination Strategy (2017-2026) focusing on achieving a malaria-free Thailand by 2024, key priorities include expansion of service coverage to at-risk population, effective surveillance system, adoption of new technology, capacity building of new staff, and integration of MP, MC and health promotion hospital (HPH). - DVBD outlined two-pronged integration approaches: transfer of malaria diagnosis and treatment activities to public hospitals (sub-district and district levels) and of vector control activities to local surveillance and rapid response team (SRRT); both with DVBD and respective PHO support | **National malaria programme:**  DDC, Global Fund management office;  DVBD, 11 ODPCs, Vector Borne Disease Center (VBDC), Vector Borne Disease Unit (VBDU), 180 MCs |
| - **Malaria post worker (MP) programme** were founded in 2004 and were managed by provincial health offices in respective provinces as part of a general health system. Many MPs were generally recruited from a pool of VHVs and/or other community-based programmes. - Supervision of MPs is conducted on a monthly basis by PHO staff who may also oversee other control activities for other diseases beyond malaria; HPH director is responsible for the supervision of VHVs under general public health system. - MPs receive introductory and refresher trainings on an annual basis; technical guidance and capacity building are provided by VBDC, while PHO facilitate training. - Community-based volunteers generally receive monthly compensation (not salary) for their volunteer work; for example, MPs receive 3,000THB and VHVs 600-1,000THB. - Accordingly to 1996 Ministry of Public Health (MoPH) Legislation on medical procedures (last updated 2013), non-medical professionals are not allowed to perform medical procedures, including blood testing, except under the supervision of medical professionals. This applied to MPs and VHVs under supervision of PHO and respectively. | **General health system:** 40 Provincial Health Offices (PHOs), 444 Health Promotion Hospitals (HPHs), 400 Malaria post workers (MPs), Village Health Volunteers (VHVs) |
| - **Civil Society Organizations (CSOs)** are sub-recipients of RAI3E grants. They also managed their own malaria programmes where they built their own health providers, volunteers and field workers, including leveraging existing providers (VHVs and MPs) in the communities to remote and hard-to-reach communities, especially along borders and forested areas home to mobile and migrant populations, illegal workers, ethnic groups, and marginalized populations. - Similar to general volunteers, CSO-based volunteers were not able to perform medical procedures. However, CSOs implemented alongside clinics (e.g. SMRU) were able to perform medical procedures and provide medical services including diagnosis and treatment. - Monthly salary and activity-based incentives were provided for workers and volunteers involved in specific programmes. | **CSOs:** Alight, International Rescue Committee (IRC), Raks Thai Foundation (RTF), Shoklo Malaria Research Unit (SMRU), Young Muslim Association of Thailand (YMAT) |
| - **Local administrative offices** (LAOs which include provincial and sub-district administrative offices) and local health network are emerging actors in the malaria elimination program whereby surveillance and malaria prevention measures - Future financing opportunities include local health funds to support disease outbreak prevention and control - 2020 Guideline for Local Administrative Offices and health network on the implementation of malaria elimination strategy - 2015 the Communicable Disease Act of the MoPH stipulates that local authorities are responsible for the surveillance and reporting of malaria cases | **Non-MoPH departments:**  Provincial Administrative Office (PAO),  Sub-district Administrative Office (SAO) |
| - **The Global Fund to Fight AIDS, Tuberculosis and Malaria (GFATM)** is the main donor that support the national program and implementing organizations - Technical assistance were provided by WHO Thailand, and other organizations that are representative in national malaria elimination management committee, including USAID, Armed Forces Research Institute of Medical Sciences (AFRIMS), United Nations High Commissioner for Refugees (UNHCR), and International Organization for Migration (IOM). | **IOs:** United Nations Office for Project Services (UNOPS), World Health Organization (WHO) Thailand, United States Agency for International Development (USAID) |

**2.1 Characteristic of malaria post worker according to RAI3E Implementation Plan.** Source: 2021 DVBD’s RAI3E Implementation Plan

| **Eligibility** | | |
| --- | --- | --- |
| 1. Age 18-55 years old 2. Receive primary education at minimum, literate, and able to communicate in local language(s) 3. Are local to, live in, or able to work in community where the post is located 4. Well-prepared for the tasks and able to maintain the role for the project duration; accepted by the community and selected by the village committee | | |
| **Roles and responsibilities** | | |
| 1. Screen malaria patients with RDT and provide treatment 2. Explain correct use and adherence to malaria treatment, and follow up with all malaria cases 3. Monitor treatment adherence and provide consultation 4. Report malaria cases onto malaria information system (MIS) within one day 5. Notify or accompany all PF cases to attend follow-up visits on Day 3, 7, 28, 42 6. Notify or accompany all PV cases to attend follow-up visits on Day 14, 28, 60, 90 7. Refer all cases with severe symptoms, pregnant women, and children under one to HPH or hospitals 8. Perform behavioral change communication (BCC) to target population including at-risk group and community members; tailor content, message, and channel to communicate regarding infection risk, asymptomatic malaria, testing and treatment, treatment adherence, follow-up attendance, and self-prevention measures including promoting use of LLIN and repellant 9. Coordinate vector-control intervention implementation e.g. coverage of LLIN distribution 10. Supervise malaria treatment 11. Perform active case detection among population with outdoor infection risk e.g. forest goers and plantation workers using RDT 12. Complete relevant reports e.g. paper-based case notification report (EPI form-1, and mHealth application to report case online onto MIS | | |
| **Medical equipment and supply** | | **Office equipment and supply** |
| - Tissue paper (roll, box) - Pill counting or splitter tray - Height and weight scale - Sterile cotton - Digital thermometer - Permanent marker - Pill brown Zip-lock bags - Max-Min Thermometer | - Ethyl alcohol 70% - Powder free latex gloves - Blood lancet - Microscope slides - Spreader slides - Infrared thermometer - Surgical mask - Bin - Bin for contaminated sharps | - Desk, chair - Cupboard(s) - Clock - Whiteboard or notice board - Office sign - Stationary |
| Note: MPs will be provided with RDTs and a cool box for storage, and with Artesunate-Pyronaridine for complicated PF cases and Dihydroartemisinic-Piperaquine (DHA-PIP) treatment for uncomplicated PF cases in Ubon Ratchathani and Sisaket. | | |
